# A Theory of Frustration and Its Effect

**DOI:** 10.1007/s10991-022-09305-7

**Published:** 2022-06-22

**Authors:** Sagi Peari, Zamir R. Golestani

**Affiliations:** University of Western, Crawley, Australia

**Keywords:** Frustration, Covid-19, Unforeseen event, Reasonable foreseeability, Frustration remedy, Unjust enrichment

## Abstract

One of the key legal questions that COVID-19 has raised relates to the status of the traditional contractual doctrine of frustration. The pandemic and the ongoing lockdowns across the globe have made it difficult for many contracts to perform. At the same time, there is a deep doctrinal and conceptual confusion with respect to the very essentials of this doctrine and its remedy - i.e., what happens after an adjudicative tribunal declares that a given contract has been frustrated. The paper offers a unified conceptual account of the frustration doctrine and claims that both the doctrine and its remedy crystallize a single unifying idea.

## Introduction

The doctrine of frustration addresses situations where contractual performance is affected by certain sudden, unexpected events. The doctrine is gaining significance due to the disruptions caused by Covid-19. The World Health Organisation declared Covid-19 a global pandemic on March 12, 2020 (World Health Organisation, 2020). Many contracts signed before that date became impossible or difficult to execute. Governmental restrictions and lockdowns heavily interfered with the availability of labour alongside causing significant disruption to the timelines for delivery of goods and services. The business efficacy in many dealings was lost. Even today, the pandemic clearly affects contracts and the expectations of commercial actors. After months of relative predictability, countries such as Australia suddenly went into tough lockdowns due to Covid-19. The successful vaccination campaigns in other countries have only proved to be a mirage of ‘back to normal’, with the advances of the ‘Delta’, ‘Delta Plus’ and ‘Omicron’ variants and the troubling scientific studies pointing to the declining effectiveness of the existing vaccines. This has led to a new wave of restrictions and lockdowns, with further disruptions to commercial activity.

However, there are other reasons that support the critical relevance of the frustration doctrine. One reason relates to the growing consumer protection legislation that interferes with the contractual terms and conditions. While the traditional common law approach honoured the ability for parties to freely structure their *force majeure* clauses (e.g., Patterson and Robertson 2020: 398–399; Treitel 1994: 415–456) - the contractual provisions that explicitly specify the nature of the unforeseen events and the applicable remedy - today, the situation seems to be changing. Consumer protection legislation maintains the ability to challenge the validity of some *force majeure* clauses.^1^ For instance, if a *force majeure* clause is a part of a standard form contract and is drafted in a one-sided way that protects the interests of only one party to the contract, the clause could be declared as void (e.g., Jane and Paterson 2020). The traditional doctrine of frustration would then step in and fill the lacuna left by the invalidated *force majeure* clause.

Finally, the relationship between the doctrine of frustration and the desired structure of the *force majeure* clauses which interact with each other remains unresolved. The contractual provisions on *force majeure* could be informed by a conceptual account of the frustration doctrine. In this way, a coherent theory of frustration could provide a *blueprint* for successful *force majeure* clauses and support practitioners tasked with drafting contracts.

This article offers a theory of contractual frustration and its remedy. It embeds the doctrine of frustration within the conceptual underpinnings of contract law and the obligations of the parties under a contract. Perhaps the two most important common law cases concerning the frustration doctrine within the 20th century, namely, the UK *Davis Contractors* 2 and the Australian *Codelfa* 3 cases, will be used as a litmus test in order to develop a conceptual framework for grasping the nature of the frustration doctrine. The article then turns to address the remedial aspect of frustration. It argues that situated within the stances of contract law theory, the doctrine of contractual frustration gives rise to a robust vision of the frustration remedy; in other words, it provides an answer to the question of what happens after the contract has been frustrated? Unfortunately, the subsisting answers to this question across common law jurisdictions are confusing and notoriously unclear. In engaging with the remedial considerations of frustration, the article focuses on the well-known UK *Fibrosa* case.^4^

The article proceeds as follows. Part II begins with some observations about the nature of contract law and contractual obligations. It then introduces the UK *Davis Contractors* and the Australian *Codelfa* cases before providing an outline of the conceptual framework for grasping the nature of contractual frustration, delineating it from alternative accounts. Part III engages with the consequences of frustration and situates this aspect of the doctrine within the suggested conceptual framework before illustrating its operation through the UK *Fibrosa* case. Finally, Part IV summarises the proposed conceptual framework of the doctrine of frustration and its remedial effect.

## Frustration: The Impairment of Contractual Performance Due to an Unforeseen Event

### Some Basic Observations of The Nature of Contractual Obligations

Contracts primarily serve as a tool for interpersonal collaboration and enforcement of legally binding promises (e.g., Fried 2014: 17, 20; Dagan and Heller 2016: 41–47).^5^ They typically involve a reciprocal (Serafin 2019)^6^*transfer of objective value* (Hegel 1820: para 63). This may be in the form of an action that one party undertakes to perform for another party (such as a delivery of a service), or a transfer of property (such as a case when ownership of an object changes hands). However, contract law is disinterested in the desired outcomes, benefits, and commercial soundness behind the decision of the contracting parties to enter into the contract. Parties may have different visions, risk allocations, commercial expectations, desirable benefits and expected enjoyments with respect to the contracts that they sign and the ‘worthwhileness’ of the contract for them.

Contract law doctrine generally prioritises the express language of the contract over the surrounding circumstances that led to its formation. Indeed, as contract law doctrine demonstrates in relation to matters such as contractual interpretation^7^ and implied terms,^8^ there seems to be an inherent tension between the two. At least on the point of departure,^9^ contract law abstracts from particular motives, desires and economic calculations that stand as the basis of the contracting parties’ actions. Instead, focus remains on the parties’ *external manifestations* that provide the ultimate basis for objective assessment of their interaction (Benson, 2019: 101–121). The express written terms and conditions of the contract alongside the ‘four corners of the contract’ represent the ultimate crystallization of this manifestation. By agreeing to the terms and conditions of the contract, the parties communicate to the external world the nature of their agreement as an exchange of obligations, which in turn involves a reciprocal exchange of something of value.

Many contracts do not work out in the manner expected by the parties at the time of formation. Many times, contracts will lead to financial losses. However, contracts cannot secure the expectations of the parties. Rather, contract law focuses on the *time of the contract formation* (e.g., Baker 1979).^10^ When parties sign a contract, they are concerned only with the explicit terms of the contract as understood within the context of the surrounding circumstances at the time.

A final point must be made regarding the nature of the rights that each party acquires under the contract. Despite some suggestions to the contrary (e.g., Benson, 2019: 4, 41, 247, 321, 358–359), the nature of these rights relates to the contractual performance of each party. This is indeed what stands at the basis of the contractual agreement (Kant 1798: para 6: 274). Consider a scenario under which I purchase a horse from my neighbour for $1,000. I acquire a right to the contractual performance of my neighbour, namely, the delivery of the horse. In the same manner, my neighbour’s right against me relates to my contractual performance of paying her $1,000. This vision of contractual rights explains the primary remedy of the contract law doctrine in the case of a breach: the remedy of expectation damages.^11^ By placing a defendant in the position he or she would have been in had the contract been performed, this remedy crystallizes the plaintiff’s loss resulting from the breach. Where a contract is breached, the plaintiff is ‘deprived’ of the performance of the defendant (Kant 1798: para 6: 274; Ripstein 2009: 107–144).^12^

Clearly, in the case of a transfer of property, the contractual rights closely interact with property rights. Returning to the example of the horse, the parties can agree that I pay the full amount of $1,000 today for delivery of the horse tomorrow. We may also agree that my neighbour will maintain the possession and ownership of the horse until its delivery. Generally, common law would honour such an arrangement; the property sits where the parties allocate it.^13^ If my neighbour breaches the agreement and does not deliver the horse tomorrow, my remedy is limited to my expectations under the contract; I cannot make an alternative property-based claim of detinue (e.g., Chambers 2019: ch 7) according to which the horse must be repossessed to me.

Armed with these basic observations about the nature of contractual obligations, attention will now shift to the doctrine of frustration and the two key decisions that illustrate its operation: the UK *Davis Contractors* (1956) and the Australian *Codelfa* (1982) cases.

### Two Cases

#### Davis Contractors (UK)

*Davis Contractors* involved a contract between a municipality and a building company under which the company was supposed to erect a number of houses within an eight-month period. However, due to a shortage of skilled labour in post-war England, the work took significantly longer than the eight-month period scheduled. The company continued working on the project without interruption for a period of 22 months.^14^ Unsurprisingly, the building company suffered a loss under this contract; while the total contract price was £94,424, the total amount of expenses related to the contract amounted to £115,233. Not only was the total contract price insufficient to cover the expenses, it made the project commercially disastrous for the builder.^15^

The company argued that the contract was frustrated due to the shortage of skilled labour.^16^ If accepted, this argument would have meant that the contract had come to a conclusion at some point.^17^ Accordingly, as the work continued on the site without interruptions, the company argued that a new contract was formed which entitled it to a reasonable and fair remuneration for the works performed after the end of the original contract; in other words, a *quantum meruit* remedy.^18^ In reply, the municipality argued that the shortage of labour did not amount to frustration.^19^ Not only was a catastrophic event missing in the factual scenario of the case, it was also hard to point to a precise clear-cut point in time within the parties’ interaction that would signify frustration.^20^ The building company simply undertook the risk of the shortage of labour. The municipality argued that a determination of frustration in these circumstances would operate to ‘justify interference with almost any commercial contract’.^21^

Their Lordships favoured the municipality’s position and made important observations on the nature of the frustration doctrine. Lord Radcliffe commented that:

frustration occurs whenever the law recognizes that *without default* of either party a contractual obligation has *become incapable of being performed* because the circumstances in which performance is called for would render it *a thing radically different* from that which was undertaken by the contract. Non haec in foedera veni. It was not this that I promised to do.^22^

Lord Radcliffe added that the doctrine of frustration is fundamentally grounded within the concept of ‘foreseeability’ of the contracting parties at the time the contract was formed. This explains why hardship or inconvenience to perform a contractual obligation falls short of the threshold required to engage the frustration doctrine.^23^ Rather, the simple point is that parties should not perform something that they could not foresee.^24^

In similar vein, Lord Somervell of Harrow commented that contractual performance is based on the contracting parties’ expectations,^25^ while Lord Reid observed that the frustration doctrine relates to the question whether the original contract can accommodate the frustrating event.^26^

#### *Codelfa* (Australia)

*Codelfa* also involved a contract between a municipality and a building company, this time concerning the construction of several railway tunnels. The agreed timeline for performance played an important role in the contract and required the contractor to complete the works within several months. This involved continuous work on the site around the clock with 3-shifts per day, 6-days per week.^27^ Although the construction work was expected to generate a considerable amount of noise, the contracting parties did not expect that the residents of the nearby neighbourhood would be successful in seeking an injunction order from a court that would restrain the permitted working hours as one of the municipality’s acts at that time granted it immunity and barred such injunctions against works conducted on its behalf.^28^

Ultimately, the contracting parties had overestimated the scope of the defence under the immunity provision. To the surprise of both parties,^29^ the local residents managed to receive the injunction order in relation to the noise and hours of work. It is not that the parties failed to consider the possibility of an injunction order. Indeed, they had. Rather, both parties operated under the assumption that it would not happen.^30^ The building company made a similar argument to the one made by the building company in *Davis Contractors*: the contract had been frustrated which should result in a reasonable and fair *quantum meruit* compensation for the works performed after the injunction order.^31^

In contrast to *Davis Contractors*, the judges accepted the frustration claim in *Codelfa*. While much of the judicial reasoning referred to *Davis Contractors*, the decision included some important observations about the nature and operation of the frustration doctrine. Thus, the judges followed *Davis Contractors* on the point that the doctrine applies to situations when the contractual performance becomes fundamentally different from the performance in a situation contemplated by the contract.^32^ At the same time, the court observed that situations when a performance of contractual obligation becomes illegal^33^ or impossible^34^ are good indicators of the frustration doctrine, but do not automatically engage it. In other words, impossibility or illegality of performance does not necessarily leads to frustration.

### The Nature of Frustration

#### The Two-Limb Structure

The *Davis Contractors* and *Codelfa* cases provide a fertile ground for grasping the normative nature of the frustration doctrine. One of the central lessons of the two decisions is that the doctrine of frustration appears to include two major limbs: (1) an unforeseeable event; and (2) that the unforeseeable event must interfere with the performance of the contract. These two limbs seem to be interrelated and yet normatively separated. First, the doctrine applies only in the case of an event that could not be reasonably foreseen by the parties at the time of contract formation. While the injunction order was not foreseeable in *Codelfa*, the shortage of skilled labour was foreseeable in *Davis Contractors*. Second, even if the event is unforeseeable, the event also *must affect* the performance of the parties. As we have seen in both cases, the event affected the scheduled timeline of the works and therefore interfered with the contractors’ performance.

It is submitted that the time of contract formation represents the focal point of the doctrine. The contracting parties reasonably anticipate a broad range of events and developments that may take place throughout the life of the contract. This may include a variety of expenses, inconveniences, price fluctuations and other risk allocations and benefit expectations. In *Davis Contractors* the parties could simply anticipate the interfering event. As Lord Reid put it: ‘[The delay] was not caused by any new or unforeseeable factor or event: the job proved to be more onerous but it never became a job of a different kind from that contemplated in the contract’.^35^ That was not the case in *Codelfa*, where the injunction order did not lie within reasonable contemplation of the parties at the time of the contract formation. While the broad range of possible risks and benefits usually are not found within the explicit contractual terms and conditions, it represents a key aspect of the background circumstances that surround the contract. These background circumstances provide the basis for the contractual agreement under which each party to the contract accepts to undertake his or her obligation.

If the background circumstances of the contract changed so dramatically that they extend beyond the reasonable expectations, risk allocations, and benefits calculated by the parties at the time of the contract formation, this defeats the very nature of the contractual obligations made by the parties.^36^ In other words, frustration does not attempt to accommodate parties’ motives and wishes at the time of contract formation. Instead, the doctrine operates to acknowledge the possibility of the existence of dramatically rare situations when one (or both) of the contracting parties must perform an obligation that could not have been anticipated when the obligation was undertaken. Unforeseeable events that interfere with the ability of the parties to perform their obligations leads to a situation where the original foundation of the contractual choice disappears.

From this perspective, the doctrine of frustration resembles the doctrine of a common mistake (e.g., Treitel, 2020: 1106–1107).^37^ Under the latter doctrine, the law provides that a contract cannot be formed if both parties to the deal make a mistake with respect to one of the key elements of the contract (e.g., Treitel, 2020: 353–389). The doctrine of frustration follows this same rationale. While in the case of a common mistake, no contract is ever formed due to the lack of a reasonable factual basis upon which the agreement has been reached, instances of frustration represent a situation when the contract *is* formed. However, similar to common mistake, at some point (more specifically, the point of the dramatic intervening event) reality pulls apart the original anticipatory basis of the contract.

The dramatic, unforeseeable change in the circumstances of the contract disempowers the parties’ choice that stands as the basis of their obligations. This is why frustration defeats the very nature of the contract and discontinues the contractual rights that each party maintains under the contract. Since the objective manifestations of the parties rely on the anticipatory basis at the time of formation, frustration breaks apart the established contractual rights and duties. The question here is whether the reasonable anticipatory basis of the contract is present. If it is not, a performance of the contract in those circumstances would mean imposing on a person to do something that he or she never agreed to.

These observations shed light on the various doctrinal aspects of the frustration doctrine. First, there exists the two-limb structure of the doctrine. The suggested vision of frustration provides a normative explanation: the unforeseen event must be so dramatic that it does not lie within the broad spectrum of possible risks and benefits calculated by the parties at the time of contract formation. The interference with the obligation of performance of one (or both) of the parties is indeed what creates the problem that the doctrine of frustration aims to address. Without a causal link between parties’ obligations and an intervening event, the performance of the contract remains untouched.

Second, and closely related, is that this vision of frustration focuses on the dramatic non-foreseeable nature of the intervening event and accepts *any interference* with contractual performance. The interference could be very serious, such as destruction of the subject matter,^38^ the illegality of performance^39^ or other impracticalities.^40^ While the degree of interference with contractual performance may indeed provide an indicative sign of frustration, *any interference* with contractual performance would be sufficient to establish frustration as long as the intervening event meets the high threshold of non-foreseeability. While in *Davis Contractors* the shortage of labour led to significant expenses of the building company, the amount of expenses itself does not affect the question whether frustration takes place or not. What matters is that the shortage of labour became, at some point, unforeseeable; if so, any interference with contractual performance would constitute frustration. The same point applies to *Codelfa*: what matters is the anticipatory basis of the injunction order, rather than the significant additional cost that the project incurred due to the frustrating event. As long as there is *some* interference with contractual performance, the second limb of the doctrine is satisfied. This vision of frustration shifts the focus from severity of the impact towards the nature of the intervening event. It limits the scope of the first limb of the doctrine and pushes forward the limits of the second.

Third, there is a question relating to a clear-cut point of the interfering event. As we have seen in Part II (B)(1) above, this was a central point in *Davis Contractors* and is a factual inquiry.^41^ The question here is whether something beyond the reasonable expectations of the parties had taken place that affected the ability of either of the parties to perform a contractual obligation. The fact that the frustrating event might be ongoing complicates matters, and yet does not affect the normative dimension of the doctrine. Clearly, the frustrating event must start at some point. In *Davis Contractors*, that could be the time when the shortage in labour commenced or at some latter point. This is up to the court to determine whether throughout contractual performance the original anticipatory basis maintained by the parties remains firm. In *Codelfa* this determination is relatively uncomplicated, with the relevant point in time of frustration was at the date of the injunction order.

The current evolving reality of the COVID-19 pandemic well-illustrates this point. The timeline between the first reports from China (December 2019) until the day when the World Health Organisation declared the coronavirus a global pandemic on March 12, 2020 (World Health Organisation, 2020) could be considered a reasonable timeline for invoking the frustration doctrine. One should suspect that contracts signed within this timeline may be frustrated by the COVID-19 pandemic. A party claiming frustration would still need to show that the unforeseeable event (i.e., the pandemic’s outbreak) impaired the performance of obligation/s under the contract (e.g., Morgan 2021). However, contracts signed after March 12, 2020 generally do not seem to be subject to frustration, as the evolving reality of recurring lockdowns, restrictions and commercial inconvenience falls within a reasonable anticipatory spectrum of risk allocation at the time of the contract formation.

Finally, there is the issue of fault. Both *Davis Contractors* and *Codelfa* stressed the point that the intervening event must not be the fault of either of the parties.^42^ The suggested understanding of frustration offers an easy explanation to this requirement. The intervening event must not to be attributed to either of the parties, not because of the nature of the fault itself, but rather due to the *foreseeability of the fault*. At the time of the contract formation the parties can reasonably foresee the possibility of a contract breach by one of the parties. In other words, the event lies within the reasonable foreseeability of the parties at the time of the contract formation, thus pre-empting the application of the frustration doctrine.

#### Clarifying the Suggested Vision of Frustration Through the Alternatives

How does the suggested vision of frustration relate to the existing conceptions of the doctrine?^43^ While it is at odds with some, it indeed clarifies others. Consider first, the accounts that we reject: (1) absolute contracts; (2) justice/fairness; (3) implied terms; (4) radically different theory; and (5) multi-factorial approach.

First, there is a historical ‘absolute contract’ approach which rejects the frustration doctrine altogether. While we embrace the current, high threshold for doctrine recognition in the courts (e.g., Treitel, 2020: 1036–1037), our conceptual account allows room for the frustration doctrine in exceptional circumstances. Second, we object to the justice/fairness justification.^44^ It says that the doctrine should be recognised to meet the requirements of justice and fairness in particular cases (e.g., Treitel, 2020: 1102) in order to ‘do justice between the parties’.^45^ We doubt this position due to its uncertainty with respect to the very question of what ‘justice/fairness’ actually means and due to its departure from a principled understanding of private law rules, principles and concepts as a reflection of the parties’ rights and duties. Third, our approach is at odds with the ‘implied terms’ understanding, under which the contracting parties implicitly agree to end their contract in the case of unusual circumstances (e.g., Treitel, 2020: 1101). If anything, frustration cases are not reconcilable with parties’ agreements. The very crux of our argument follows this logic: the doctrine of frustration is conceptually coherent precisely because the agreement to undertake the obligations under the contract is not present.

Fourth, the suggested account is at odds with the ‘radically different theory’ of the doctrine which focuses on the parties’ performance (e.g., Treitel, 2020: 1103–1104). We would argue that this theory mischaracterises the very nature of the doctrine by overlooking the first limb and focusing too heavily on the second. Instead of focusing on the nature of the event that leads to *any* interference with performance, the ‘radically different theory’ is incorrectly preoccupied with the examination of the question to what degree (i.e., examination of the magnitude) the actual performance departs from the original performance. The suggested normative structure of frustration turns away from such an inquiry.

Consider the *Tsakiroglou* case^46^ which concerned the prolonged and expensive shipping of goods through the Cape of Good Hope as a result of the closure of the Suez Canal.^47^ Our analysis of this case would depart from the judicial reasoning which considered the difference between the original performance (i.e., shipping through the Suez Canal) and the new one (i.e., shipping through the Cape of Good Hope). The court focused on the degree of impact that the new performance requirements had on the parties in terms of their expenditures and the shipping timeframe.^48^ We would argue that the court did not consider the following critical point in this case: the foreseeability of the war between Israel and Egypt that led to the Suez Canal closure (Encyclopedia Britannica, 2021). Had the court decided that this war could not have been reasonably anticipated, *any* change to the parties’ performance under the contract would have constituted frustration.^49^ The reason for this absolute position lies in the very nature of contractual relations; once the frustrating event takes place, any change in performance means that a party (or parties) is asked to perform something that they did not agree to.

The recent Australian *Happy Lounge* case^50^ well-illustrates this point. It involved a sale of a bar which sold food, alcoholic beverages and provided live music entertainment. The contract was signed on February 16, 2020. On March 23, 2020, the local authorities issued a health emergency decree which prohibited the operation of such businesses. While the buyer invoked the ‘frustration’ argument, the court held that the decree did not deprive the buyer of a ‘substantial’ benefit of the contract.^51^ We would argue that the court should have accepted the frustration argument in this instance. In similar vein to the observations on the *Tsakiroglou* case, the court’s analysis of the foreseeability of the governmental decree and the magnitude of the impact of that decree could have been challenged. The focus should have been on the *foreseeability* of the pandemic and the subsequent question of whether the pandemic in any way affected the performance. Given the timeline of contract formation and performance, the frustration argument in this case should have been successful.

Finally, our argument is inconsistent with the multi-factorial approach which suggests incorporating a broad range of factors to be considered and weighed against each other.^52^ The suggested unifying normative basis would challenge the multiplicity of often self-contradictory vectors. While it places some of the factors favoured by the multi-factorial approach (such as parties’ reasonable expectations as to risks and the nature of the intervening event) under a single basis, we join the literature that doubts the adequacy of this multilayered justification to frustration (e.g., Roberts 2021).

Alongside the rejection of the above alternatives, we would argue that the suggested account clarifies other conceptual visions of frustration: (1) the foundation; and (2) the construction theories of frustration. First, consider the remarks made by Lord Haldane in the *Tamplin* case^53^ where his Lordship stated that ‘the occurrence itself may be of a character and extent so sweeping that the foundation of what the parties deemed to have had in contemplation has disappeared, and the contract itself has vanished with that foundation.’^54^ While this statement links the conceptual bases of the doctrine to the very foundations of the contract, the subsequent literature (e.g., Treitel, 2020: 1102–1103) and case law have perceived it as notoriously vague and amorphous. In *Canary*, Smith J commented on this point, stating that ‘[a]lthough attractively phrased, this theory [the foundation theory] is no more than a form of words, with no clear meaning behind it.’^55^

We would argue that the suggested account sheds normative light on the foundation of contractual frustration. The objective notion of agreement is the one that stands as the basis of every contract. If the supervening circumstances repeal the very basis upon which the parties reach the agreement with respect to their obligations, this notion challenges the very foundation of the contract. Once the high threshold of unforeseeability is met, any deviation from the original obligation leads to frustration of the contract.

Second, a related point applies to the ‘true construction’ theory of frustration. This justification focuses on the construction of the force majeure clauses and contemplates whether a given clause covers the occurrence of the frustrating event. Some of the literature has correctly pointed out that the construction justification cannot provide a normative justification to frustration.^56^ By focusing on the *force majeure* clauses,^57^ it tackles the question of their proper interpretation. However, the ‘construction’ theory of frustration does not really explain the very nature of frustration in any detail.

The conceptual account of frustration matters. It is important for grasping the nature of the doctrine and for contemplating its future development. One must carefully delineate cases that constitute frustration and those cases that do not. Furthermore, as we will see in the next part, this conceptual account also aims to shed light on the other side of the doctrine, namely, its remedial consequences.

## The Frustration Remedy

### The Uncertainty

What happens after a contract is frustrated? This question remains highly debatable within the common law literature and subject to notorious confusion (e.g., Stewart and Carter 1992; Carter 2018: 773–792). It is not that the common law jurisdictions have not tried to address the confusion; in fact, explicit attempts have been made to address it. In 1943, the UK Parliament enacted the *Frustrated Contracts Act* 58 to address the problem. Several Australian jurisdictions followed this path by enacting distinct legislative schemes, namely, the *Frustrated Contracts Act 1978* (‘New South Wales Act’),^59^ the *Frustrated Contracts Act 1988* (‘South Australian Act’),^60^ and part 3.2 of the *Australian Consumer Law and Fair Trading Act 2012* (‘Victorian Act’).^61^ In all other Australian States and Territories, the common law as laid out in *Fibrosa* continues to govern the consequences of frustration (Barker and Grantham 2008: 253). Unfortunately, despite the various legislative attempts, common law jurisdictions failed to reach a consensus with respect to appropriate remedial response to frustration and allocate this response within the conceptual stances of the contract law doctrine (e.g., Stewart and Carter 1992: 69–79).

The UK case of *Fibrosa v Fairbairn* 62 is a good starting point to illustrate the failure of contemporary jurisprudence to coherently address the question of the remedial effect of frustration. This case involved a Polish company that agreed to purchase machinery from a UK company, with delivery to be made in Poland. After the Polish company had made a sizeable deposit, Nazi Germany invaded Poland, making performance impossible and frustrating the contract. Not a single unit of machinery had been delivered and the contract itself was silent on the point of frustration and the remedial effects of frustration. The UK company relied on precedent established in the previously decided *Chandler* case which asserted that the ‘loss lies where it falls’.^63^ Furthermore, they argued that it incurred significant expenses related to the production of the machines.

Their Lordships overruled the *Chandler* precedent and decided that the Polish company should receive its deposit back. Somewhat vague and diverse reasoning lay at the basis of this decision. Lord Atkin talked about ‘most people, whether laymen or lawyers’ who would think ‘the buyer ought to get his money back…’,^64^ favouring a rule that would ‘enable a man who has paid money and received nothing for it to recover the money…’^65^. Lord Macmillan commented about ‘equitable adjustment between the parties’^66^ and that anything else would ‘fall short of complete justice’.^67^ Lord Wright mentioned the doctrine (unrecognised at that time) of unjust enrichment as a possible conceptual framework for grasping the nature of frustration remedy.^68^ Other justifications referred to the conditional nature of the deposit. As Lord Wright put it: ‘The payment was originally conditional. The condition of retaining it is eventual performance. Accordingly, when that condition fails, the right to retain the money must simultaneously fail’.^69^ Along the same lines, Lord Roche made mention of the ‘provisional nature’ of the deposit made by the Polish company.^70^

The *Fibrosa* case set a harsh precedent of a ‘total failure of consideration’ according to which the restitution of the payments made under a frustrated contract would only be possible when the party who made the payment can demonstrate that he or she has not received any consideration in return for the payment.^71^ In *Fibrosa*, the Polish company did not receive any consideration for its advanced payment, which indeed justified the restitution of the deposit. Since not a single machine was received, the judgment went for the plaintiff. Had the Polish company received even one machine, this would have precluded the restitution of the deposit.

The doctrine of a ‘total failure of consideration’ has been heavily criticised on the grounds of its rigidity (e.g., Birks 1996; Edelman 1997). Some favour a possible re-interpretation of the doctrine to include a ‘partial failure’ of consideration as well (e.g., Wilmot-Smith 2013). The above-mentioned legislative acts in the UK and some of the Australian jurisdictions were aimed precisely at overriding the harshness of the ‘total failure of consideration’ doctrine. The legislation in Australia and the UK favours restitution of monetary payments made under frustrated contracts regardless of whether the failure is ‘full’ or ‘partial’.^72^ These legislative pieces have incorporated a wide range of open-ended provisions that provide courts with the discretion to also take the expenses incurred by the parties into account^73^ or to adjust the entire award with seemingly unbridled discretion in order to promote ill-defined notions of ‘justice’.^74^ Further, the scope of application of these provisions extends beyond monetary transfers and is potentially applicable to situations involving contracts for provisions of services.

Despite the efforts to address the deficiencies of *Fibrosa*, the current legislative provisions in Australia and the UK concerning the remedial aspects of frustration evidently require a significant overhaul. These provisions grant almost absolute discretion to the judges to consider the cases as they wish, on a case-by-case basis. Furthermore, it would appear that each legislative piece relies on a different normative vision of frustration remedies, which explains the significant language discrepancy used in each of the provisions. To no one’s surprise, this unprincipled, no-rule approach attracted much criticism, creating doubt regarding the desirability of the legislative intervention in the first place (Stewart and Carter 1992: 108). Accordingly, it is little wonder that these statutes are seldom utilized in the UK^75^ and Australia.^76^

In recent decades, the advancing law of ‘unjust enrichment’ has tried to accommodate the remedial aspect of frustration under its auspices. As we have seen above, some of the comments made by the judges in *Fibrosa* indeed supported this direction. At the basis of the law of unjust enrichment lies the fundamental claim that an ‘unjustified’ transfer of something of objective value from the plaintiff to the defendant triggers a restitution of that value (Birks 1989: 16–18). The defendant should not unjustly benefit at the expense of the claimant.

This principle has received official recognition in the UK *Lipkin Gorman* case.^77^ Australian jurisprudence struggles and hesitates on whether to adopt this law as a separate ground of liability in private law.^78^ In relation to the remedial aspect of frustration, unjust enrichment scholars have argued that it would be ‘unjust’ for the English company in *Fibrosa* to retain the deposit (e.g., Edelman and Bant 2016: 255–256). Furthermore, the expenses incurred by the English company could also be taken into consideration in the calculus of the parties’ entitlements as those expenses could be viewed as part of the unjust enrichment doctrine titled ‘change of position’ (e.g., Bant 2008: 243–249). According to this doctrine, the court should consider the fact that the defendant relies on the benefit received and spends it. In some circumstances, according to unjust enrichment scholars, such reliance is legitimate and should be taken into the consideration for restitution purposes (e.g., Bant 2008: 162–163; Goff and Jones, 2016: 776–777). In this way unjust enrichment claims to explain the remedial nature of frustration and its various aspects.

The difficulty with the ‘unjust enrichment’ foundation of the frustration remedy lies in the shaky foundations of the unjust enrichment project itself. Consider each one of the four key elements of the analytical framework of the principle: (1) the defendant’s enrichment; (2) the enrichment is at the expense of the claimant; (3) the enrichment is unjust; (4) availability of applicable defences to the defendant.^79^

First, consider the *enrichment* element that has been characterised by many unjust enrichment supporters as referring to the notion of a ‘transfer of value’ (e.g., Weinrib, 2020: 171–174; Birks 1989: 23).^80^ The notion of ‘value’ seems to be important and clearly plays a central role in the contract law category: any contract for the transfer of property or delivery of services involves the transfer of something of value. However, one can challenge the question of the significance of the transfer of value outside of contract law. Further, the notion of ‘transfer of value’ is hard to accommodate with a central pillar of private law that relates to the parties’ rights and duties (e.g., Stevens 2018: 583–584, 590; Nadler 2008: 248–261). If it could be argued that contract law protects claimants’ right of contractual performance and that tort law protects claimant’s right to bodily integrity and property (e.g., Ripstein 2016), which right does the transfer of value protect?

Second, the notion according to which enrichment is *at the expense* of the claimant seems to simply follow the general insight of private law that situates the litigating parties’ rights and duties in a relational manner. This notion reflects the bipolar relationship between the litigating parties. However, this relational aspect of the unjust enrichment doctrine appears to simply reflect the obvious nature of private law; it does not provide a normative uniqueness to the structure of unjust enrichment. One of the immediate conceptual questions relates to the exact nature and magnitude of the required interaction between a particular claimant and a particular defendant (e.g., Smith 2018: 98–100).

Third, the *justice* element of unjust enrichment requires pause. Clearly, all normative elements of the private law liability should reflect the needs of justice. Unsurprisingly, unjust enrichment supporters have not tried to make an example of interpersonal relationships that are ‘just’. Rather, the unjust enrichment movement has referred to a broad range of so-called ‘unjust factors’ (e.g., Goff and Jones, 2016: ch 5; Edelman and Bant 2016: 199 − 130; Burrows 2011:86–87) that satisfy the ‘justice’ element in the unjust enrichment formula. The shift towards ‘unjust factors’ raises serious conceptual doubts regarding the question of whether there exists a single unifying thread that may be said to link the unjust factors together (e.g., Hedley 2019; Watts 2016). A failure to provide a unifying theory applicable to the unjust factors suggests that the unjust enrichment framework could not claim a normative independence.

Finally, there is a conceptual difficulty related to the various *defences* developed by unjust enrichment scholars. Take for example, the above-mentioned ‘change of position’ defence which states that in certain circumstances the defendant may limit their total restitutionary liability. A situation when the defendant acts in good faith, relies on the enrichment and ‘spends’ the benefit, represents a paradigmatic example of this defence. However, the conceptual difficulty with this defence is that it seems to be completely unrelated to the interpersonal relationship between a particular defendant and a particular claimant (e.g., Grantham 2018: 5). The fact, for example, that the defendant relied on the claimant’s benefit and spent it, is not a factor which relates to the claimant. This not only seems to be unfair to the claimant, but also challenges the conceptual coherency of this doctrine.

Unsurprisingly, the law of unjust enrichment has attracted a high volume of criticism, pointing to the lack of historical, conceptual and doctrinal coherency (e.g., Jansen 2016, Gallagher et al. 2020).^81^ The problem with the law of unjust enrichment is a serious one. One of the leading private law commentators went as far as to characterise the present status of unjust enrichment doctrine as a ‘Disaster’ (Stevens 2018).^82^ Given the growing volume of criticism mounted against the unjust enrichment project, the true basis of the remedial angle of the frustration doctrine must lie elsewhere.

### Property, Not Contract or Unjust Enrichment

Given the law of unjust enrichment does not seem to provide an evident solution to the remedial aspect of frustration, the contract law and property law doctrines are the next possible candidates. First, consider the contract law doctrine. In *Davis Contractors*, the municipality made the promise to pay; the building company made the promise to erect the buildings. The reciprocal obligations of each party hinges on the actual terms and conditions of the contract, as pointed out by the Lord Reid.^83^ However, as we have seen in Part II (C), frustration involves a dramatic change in the circumstances that goes beyond the reasonable range of risks and benefits at the time of the contract formation. If, and only if, the event falls outside of the parties’ reasonable expectations at the time of contract formation are their contractual rights ineffective from the point of the intervening event. The duty to perform a contractual obligation ends with this event, as frustration undermines the very foundation of the agreement to perform an obligation. From this perspective, the comments made in the case law about the end of the contract and unenforceability of the parties’ future obligations^84^ all comply with the underpinnings of contract law. From the standpoint of *contract law theory*, the loss lies where it falls. The *Chandler* case was correctly decided, at least from the standpoint of contract law.

However, in this article we do not follow the path of unjust enrichment scholars who complain about the shortfall of the traditional private law doctrine to accommodate possible restitution remedies in the case of contract frustration (e.g., Burrows 2011: 35–43; Virgo, 2015: 51–58). While contract law doctrine indeed does not accommodate such remedies, a reference to property law could do so without embracing the ‘unjust enrichment’ project and the accompanying criticism raised against its conceptual and practical foundations. In other words, it is submitted that while contract law does not help with remedial aspects of frustration, *property law* does.

The difficulty in accommodating a possible proprietary restitution remedy in the context of frustration cases lies in the traditional reluctance of the common law doctrine to perceive the concept of ‘money’ within the stances of property law. However, things seem to be clearly moving in another direction (Fox 2008: 18–24, 31–33, 34–38, 42–50, 73).^85^ Consider the factual scenario in the *Fibrosa* case. Without delving into the question whether this scenario meets the suggested two-limb structure of the frustration doctrine in the first place (most likely it does not, as the Nazi invasion of Poland could reasonably be anticipated – e.g., Clavin 2015: 7, 34), from the standpoint of the remedial level of frustration the *contractual obligations* of the parties ended with Nazi’s invasion of Poland that interfered with the performance of the obligations. However, the *proprietary allocation* of the parties’ rights and duties deserves attention. This requires an attentive review of the terms and conditions of a given contract. In some cases, the parties may explicitly decide that the property in goods (or money) passes hands with the contract formation. In other cases, they may say it does not.^86^ As we have seen in Part II(A), the common law courts accept the notion that the property sits according to parties’ allocation.

In *Fibrosa*, the Polish company could have invoked its restitutionary right in the deposit by simply claiming its proprietary interest in it. In *Fibrosa* Lord Wright commented along these lines:

The claim for money had and received is not, in my opinion, a claim for further performance of the contract. It is a claim outside the contract. If the parties are left where they are, one feature of the position is that the one who has received the prepayment is left in possession of a sum of money which *belongs to the other*.^87^

These comments echo the point that the Polish company’s entitlement to restitution does not hinge on the contractual obligation of the UK company. Nor does it relate to the law of unjust enrichment. Rather, the claim of the Polish company is a proprietary one.

Nevertheless, there is the question of the proprietary allocation in cases when the contract is silent on this matter. Things become difficult in situations when the parties do not explicitly specify the timing of the property transfer in the contract. The following presumptive default rule could be established. According to this rule, the property does not pass until the buyer of a property pays the full amount of the payments. The normative grounds of this presumptive rule relate to the very nature of the contract as a transfer of values and exchange of contractual obligations.^88^ Consider the horse example under which I purchase a horse from my neighbour for $1,000 that is scheduled to be delivered tomorrow. This default rule says that my payment today of the $1,000 means that the property title in the horse moves to me immediately with contract formation and before taking possession. The situation would be different if I pay any amount less than $1,000.

There are several supportive statements of this ‘proprietary’ vision of the remedial aspects of frustration in the case law and the literature. Beyond the above-mentioned observations by Lord Wright,^89^ the comments about the ‘conditional’ nature of the Polish company’s deposit^90^ are also helpful. In similar vein, some of the unjust enrichment scholars have adopted the ‘condition’ language (e.g., Edelman and Bant 2016: 252, 267; Wilmot-Smith 2013: 415; Virgo, 2016: 308; Maher 2004: 97 − 8) as a possible justification of the ‘just’ nature of restitution. The suggested approach here goes one step further in this direction: the UK company should not hold the deposit because it was ‘conditioned’ on the delivery of the machines. Rather, the UK company should return it because it *belongs to* the Polish company.

There are several implications of the suggested approach. First, it draws a sharp line between those contracts that involved a transfer of property (whether goods or money) and those contracts that involve a performance of an action for another party. Proprietary restitution could only be relevant in the former case. Contracts concerning the performance of an action for another person may involve an improvement of a property or delivery of a service. Nevertheless, those contracts do not involve a transfer of *proprietary rights* that could potentially (and subject to property allocation) trigger restitution. In building works and provision of services, the frustration ends the contract without further restitutionary actions.

This means, for example, that the extra work in *Davis Contractors* and *Codelfa* should not entitle the contractors to the *quantum meruit* remedy,^91^ unless the contractors can show that a new contract is formed based on the parties’ conduct. True, there is a transfer of value between the parties in the case of the building works. However, one could argue that (and in contrast to unjust enrichment scholars) the transfer of value *alone* does not trigger a liability in private law. Unless the contractual terms and conditions breaks the contract into independent parts (i.e., a severable contract) or a new contract is formed,^92^*contract law* does not justify the contractor’s remuneration for unsolicited services performed. As no property passed hands, property law provides a similar negative answer.

Secondly, the suggested vision the frustration remedies denies the relevancy of the various expenses that the parties incurred prior to contract frustration. Recall that in *Fibrosa*, the UK company referred to its expenses related to the production of the machines that were incurred prior to contract frustration. However, similar to the notion of ‘transfer value’, the occurrence of expenses alone does not establish the liability of either of the parties. The incurred expenses belong to the contract law doctrine, concerned as it is with the parties’ reasonable allocation of risks and benefits, which, as we have seen, lies at the basis of the contract and are irrelevant after frustration of the contract. 93

Thirdly, the suggested approach rejects the ‘total failure of consideration’ doctrine. Rather than looking at the amount of value exchanged between the parties, it looks at the very nature of this value and allocates it within the contractual and proprietary entitlements of the parties.^94^ These are considerations that should guide the courts in instances of contract frustration, rather than considerations of obscure justice and no-rule, flexible approaches adopted throughout the various legislative provisions, which have aimed to provide coherency to the law and disarm the stringency of the *Fibrosa* rule, but perhaps have achieved the opposite.

Finally, it could be argued that the suggested vision of frustration remedies prioritises legal certainty and predictability over considerations of flexibility and fairness in particular cases. By sharply dissecting between proprietary and service-based claims and rejecting restitution of expenses incurred prior to frustration, we do not deny that. However, what does justice mean in a given case? Some would argue that the values of certainty and predictability epitomise ‘justice’ (e.g., Radbruch, 1946: 7). The analysis of the *Fibrosa* case by the unjust enrichment scholars themselves perhaps reveals the disagreement over the concept of ‘unjust’: some argue that the English company should not have returned the deposit due to the expenses incurred related to machine production (e.g., Birks, 1989: 231); others say that *Fibrosa*’s justice requires restitution.^95^ The alternative and principled approach to frustration’s remedies, which is based on the nature of the parties’ rights involved, seems to be preferred.

## Conclusions

The suggested vision of frustration clarifies the normative boundaries of the doctrine and offers a framework for its understanding. We envision a two-limb structure of the frustration doctrine that pushes each one of the limbs in a different direction. On the one hand, it favours a fairly high threshold for grasping the nature of the frustrating event. This bar is high, very high. It requires demonstrating that the frustrating event could not be reasonably foreseen by the parties at the time of the contract formation. On the other hand, our accounts adopt a fairly liberal vision of the second limb of the doctrine. Any interference with contractual performance of the parties would meet the requirements of this limb.

The article also situates the remedial aspect of frustration within the stances of the suggested argument. Frustration pulls apart the very foundation upon which contractual obligations are made. While the underpinnings of contract law indeed do not provide a remedy for restitution of benefits conferred under the contract, properly understood property law doctrine can invoke restitution in appropriate cases. Accordingly, we reject the relevance of expenses made by the parties prior to the frustration event and draw a sharp line between contracts involving transfer of property and other contracts.

The times of COVID-19 underlie the significance of the frustration doctrine and its effects. This article has offered a coherent conceptual foundation that grounds it in the underpinnings of private law categories and situates it within the parties’ entitlements. It has rejected the present path of no rules, case-by-case approach, or a reference to the amorphous concept of ‘justice’. This position indeed aligns with the general vision of contract that underlies its institutional significance to support interpersonal relationships through providing contracting parties legal certainty and predictability in planning their future affairs.

## Footnotes

1 * Senior Lecturer in Private and Commercial Law, University of Western Australia. ** JD student, University of Western Australia. Unfair Contract Terms Act 1977 (UK); Sects. 23–28, Schedule 2, Competition and Consumer Act 2010 (Cth) (Australia).

2
*Davis Contractors Ltd v Fareham Urban District Company* [1956] AC 696.

3
*Codelfa Construction Pty Ltd v State Rail Authority of NSW* (1982) 149 CLR 337.

4
*Fibrosa v Fairbairn* [1942] AC 32.

5 Fried characterises a contract as ‘a kind of moral invention’ and as providing ‘free individuals a facility for extending their reach by enlisting the reliable collaboration of other free persons’.

6 As Serafin demonstrates, the traditional contract law doctrine vehemently separated itself from the law of gifts.

7 See e.g. *Investors Compensation Scheme Ltd V West Bromwich Building Society* [1997] UKHL 28.

8 See e.g. *BP Refinery Pty Ltd v Hastings Shire Council* (1977) 16 ALR 363.

9 See e.g. *Marks and Spencer plc v BNP Paribas Securities Services Trust Company (Jersey) Limited and another* [2015] UKSC 72.

10 See also *Canary Wharf (BP4) T1 Ltd v European Medicines Agency* [2019] EWHC 355 (Ch) at [31].

11
*Robinson v Harman* (1848) 1 Ex Rep 850.

12 One could argue that the remedy of specific performance would better and more directly crystallize the notion according to which the rights acquired under the contract relate to contractual performance. However, this remedy frequently requires supervision and monitoring by the courts, what complicates the contract enforcement mechanism and therefore produces uncertainty. This indeed explains the priority of expectation damages over specific performance. See e.g. Benson (2019: 265–274).

13
*Aluminium Industrie Vaassen BV v Romalpa Aluminium Ltd* [1976] 1 WLR 676; Article 17, Sale of Goods Act, 1979 [UK]; Article 22, Goods Act 1958 Vic (Australia). Even fierce opponents of this arrangement strictly limit their objection to the specific context of secured transactions, without challenging the parties’ autonomy within the context of property law. See e.g. Wappett and Duggan (2019).

14
*Davis Contractors* [1956] AC 696, 700, 717.

15 Ibid at 715, 722.

16 Ibid at 704, 713.

17 Ibid at 706 (As the company put it, ‘There must be some stage at which prolongation of the time produces frustration.’).

18 Ibid (‘Once there was a frustration the whole contract had gone and the respondents [the municipality] could have ordered the appellants [the building company] off the site.’)

19 Ibid at 711.

20 Ibid at 717.

21 Ibid at 709.

22 Ibid at 729 (emphasis added).

23 Ibid (‘… even so, it is not hardship or inconvenience or material loss itself which calls the principle of frustration into play. There must be as well such a change in the significance of the obligation that the thing undertaken would, if performed, be a different thing from that contracted for.’).

24 Ibid at 731: (‘…the cause of the delay was not any new state of things which the parties could not reasonably be thought to have foreseen.’)

25 Ibid at 733.

26 Ibid at 721 (‘The question is whether the contract which they [the parties] did make is, on its true construction, wide enough to apply to the new situation: if it is not, then it is at an end.’)

27
*Codelfa* 149 CLR 337, 381, 412.

28 Ibid at 411–412.

29 Ibid at 413.

30 Ibid at 376, 419.

31 Ibid at 414.

32 Ibid at 383 (‘Performance by means of a two-shift operation, necessitated by the grant of the injunctions, was fundamentally different from that contemplated by the contract.’).

33 Ibid at 422, 398.

34 Ibid at 380.

35
*Davis Contractors* [1956] AC 696, 724.

36 Cf *British Movietonews Ltd v London and District Cinemas Ltd* [1951] 2 All ER 617, 625 (‘they [the parties] never agreed to be bound in a fundamentally different situation which has now unexpectedly emerged, the contract ceases to bind at that point…’)

37 See also *Codelfa*, 149 CLR 337, 381.

38 See e.g. *Taylor v Caldwell* [1863] 3 B & S 826.

39 See e.g. *Zinc Corp Ltd v Hirsh* [1916] 1 KB 541. For a recent defence of this reading of a unifying basis of the frustration events, see Roberts (2021).

40 See e.g. *Jackson v Union Marine Insurance* (1874) 10 Common Please 125.

41 Lord Reid expressed this point in the following way: ‘As and from such and such a date, at latest, the contract ceased to bind the parties.’ *Davis Contractors* [1956] AC 696, 723.

42
*Davis Contractors* [1956] AC 696, 729; *Codelfa* 149 CLR 337, 383. See also *Fibrosa* [1942] AC 32, 58.

43
*Canary*, [2019] EWHC 355 at [21]- [46]; *Davis Contractors* [1956] AC 696, 714–716, 719–722, McNair 1940; Treitel, 2020: 1100–1107.

44
*Canary* [2019] EWHC 355 at [22], [25].

45 Ibid at [25].

46
*Tsakiroglou & Co., Ltd v Noblee & Thorl G.M.B.h.* AC 93 [1961] 2 All ER 179.

47 Ibid at 181, 185.

48 Ibid at 184, 186–187, 192.

49 It is likely that in many cases of minor departures from the original performance, the contractual parties will choose to continue their contract and therefore will create a new contract through conduct. However, our point here is a conceptual one: once the event meets the (very) high threshold of frustration, any deviation from the original performance leads to frustration.

50
*Happy Lounge Pty Ltd v Choi & Lee Pty Ltd and Anor* [2020] QDC 184.

51 Ibid at [34].

52 See e.g. *Edwinton Commercial Corp v Tsavliris Russ (Worldwide Salvage and Towage) Ltd (Sea Angel)* [2007] EWCA Civ 547, [2007] 2 Lloyd’s Rep 517; *Canary*, [2019] EWHC 355 at [39]; Treitel, 2020: 1038.

53
*Tamplin SS Co Ltd v Anglo-Mexican Petroleum Co* [1916] 2 AC 397.

54 Ibid at 406.

55
*Canary*, [2019] EWHC 355 at [26].

56 Ibid (‘…whilst the true construction of the contract may be relevant to the question of frustration, it is not *of itself* the test *for* frustration.’) (emphasis in original). Also commenting that ‘even construction has its limits when faced with extreme and unforeseeable supervening events.’ Ibid.

57 The question of the proper interpretation of the force majeure clauses in light of the suggested vision of frustration would require independent treatment. However, we would argue that our account is fully consistent with the current ultra-conservative construction of the *force majeure* clauses (See e.g. *Channel Island Ferries Ltd v Sealink UK Ltd* [1988] 1 Lloyd’s Rep 323). Such a restrictive construction underlies the point that the contractual doctrine aims to ensure the validity of the foreseeability basis upon which the parties undertook to perform their obligations.

58
*Law Reform (Frustrated Contracts) Act 1943* (UK).

59
*Frustrated Contracts Act 1978* (NSW) (‘NSW Act’).

60
*Frustrated Contracts Act 1988* (SA) (‘SA Act’).

61
*Australian Consumer Law and Fair Trading Act 2012* (Vic), pt 3.2 (‘Victorian Act’). The Victorian Act replaced Part 2 C of the *Fair Trading Act 1999* (Vic) which itself replaced the *Frustrated Contracts Act 1959* (Vic).

62 [1942] AC 32.

63 Chandler v Webster [1904] 1 KB 493.

64
*Fibrosa* [1942] AC 32, 50.

65 Ibid at 55.

66 Ibid at 58.

67 Ibid at 59.

68 Ibid at 61 (‘It is clear that any civilized system of law is bound to provide remedies for cases what has been called unjust enrichment or unjust benefit, that is to prevent a man from retaining the money of or some benefit derived from another which it is against conscience that he should keep.’).

69 Ibid at 65.

70 Ibid at 75.

71 Ibid at 55–57, 61.

72 UK Act s 1(2); NSW Act s 12; SA Act s 4(1); Victorian Act s 36(1).

73 UK Act s 1(2); Victorian Act s 37.

74 NSW Act s 15(1); SA Act s 7(4).

75 See e.g. *BP Exploration Co (Libya) v Hunt (No 2)* [1979] 1 WLR 783, 798 (‘*BP Exploration*’) which was subsequently affirmed on other grounds in *BP Exploration Co (Libya) v Hunt (No 2)* [1981] 1 WLR 232 and *BP Exploration Co (Libya) v Hunt (No 2)* [1983] 2 AC 352; *Gamerco SA v ICM/Fair Warning (Agency) Ltd* [1995] 1 WLR 1226.

76 See e.g. *Ardee Pty Ltd v Collex Pty Ltd* [2001] NSWSC 836; *McRoss Developments Pty Ltd v Caltex Petroleum Pty Ltd* [2004] NSWSC 183.

77
*Lipkin Gorman v Karpnale Ltd* [1988] UKHL 12.

78 See *Equuscorp Pty Ltd v Haxton* (2012) 246 CLR 498, 516; Barker (2020: 922–928).

79 See e.g. *Banque Financière de la Cité v Parc (Battersea) Ltd* [1999] 1 A.C. 221, 227, 234.

80 See also *Investment Trust Companies (in liquidation) v HMRC* [2017] UKSC 29 at [43] (Lord Reed).

81 The US courts also traditionally disfavour the unjust enrichment principle. See e.g. Langbein (1998:61) (describing the American landscape of unjust enrichment as representing a situation like ‘…a neutron bomb has hit the field—the monuments have been left standing, but the people have been killed off.’). See also Burrows (2020: 294–301).

82 Along similar lines, Lionel Smith targets the present status of the unjust enrichment project due to the failure of this project to explain a simple case of a resident living on the top floor of an apartment who benefits from the fact that the lower-level resident is heating her apartment. See Smith (2018: 98–100). Indeed, this simple example against unjust enrichment underlies the fatal point against unjust enrichment according to which private law liability cannot be solely based on the notion of transfer of value.

83 Davis Contractors [1956] AC 696, 721.

84 As Lord Radcliffe put it in the Davis Contactors: ‘The theory of frustration belongs to the law of contract and it is represented by a rule which the courts will apply in certain circumstances for the purposes of deciding that contractual obligations, ex facie binding, are no longer enforceable against the parties.’(Ibid at 727).

85 In his works, Professor Fox demonstrates doctrinal and conceptual fallacy embedded in the artificial distinction between corporeal and incorporeal mediums of money.

86 Cf Lord Wright’s replicas in Fibrosa: ‘The principles [restitution of the payment in the case of a total failure of consideration], however, only apply where the payment is not of such a character that by the express or implied terms of the contract it is irrecoverable even though the consideration fails.’ *Fibrosa*, [1942] AC 32, 67.

87 Ibid at 69–70 (emphasis added). For somewhat a related argument (albeit made in the context of restitution of mistaken payments), see Jaffe (2013).

88 While we note that Article 18 (1) of the Sale of Goods Act, 1979 [UK] seemingly runs against this presumption, traditionally it does not apply to the context of transactions involved a transfer of money. For discussion of this point, see Fox (2008: 87).

89
*Fibrosa* [1942] AC 32, 61.

90 Ibid at 65.

91 Cf Mann v Paterson Constructions Pty Ltd [2019] HCA 32.

92 The unjust enrichment doctrine of ‘free acceptance’ according to which the services should be ‘accepted’ by the defendant follows precisely this point about the contractual foundation of this doctrine. What triggers restitution here is not the fact that the defendant ‘unjustly’ enriched at the expense of the claimant, but the notion that a *new contract* has been formed between the parties. For the discussion of this point, see Priel (2012), Burrows (2019: 536–541). Accordingly, this point clarifies our position with respect to cases where the contracting parties continued to perform their duties after the occurrence of the frustrating event (such as the case in *Codelfa* and *Davis Contractors*) and those cases when the parties discontinue such performance (as in *Fibrosa*). Our approach does not delineate between the two scenarios. When the parties choose to continue perform their obligation may constitute a formation of a new contract, as indeed was argued in *Codelfa* and *Davis Contractors*. This, however, does not change the conceptual nature of frustration.

93 This position follows the general hostility of the common law doctrine towards the protection of the ‘reliance’ interest of the parties, as opposed to the expectation interest. For the discussion of this point, see eg McLauchlan (2007).

94 Respectively, we reject the ‘total failure of consideration’ justifications of the frustration doctrine. See *National Carries v Panalpina*, [1981] 1 AC 675, 687, 702; Treitel, 2020: 1104.

95
*Fibrosa* [1942] AC 32, 59.
